# Myopia is predominantly genetic or predominantly environmental?

**DOI:** 10.1111/opo.13464

**Published:** 2025-03-03

**Authors:** Virginie J. M. Verhoeven, Ian G. Morgan, Jeremy A. Guggenheim

**Affiliations:** ^1^ Department of Ophthalmology Erasmus MC, University Medical Center Rotterdam the Netherlands; ^2^ Department of Clinical Genetics Erasmus MC, University Medical Center Rotterdam the Netherlands; ^3^ Research School of Biology Australian National University Canberra Australian Capital Territory Australia; ^4^ Zhongshan Ophthalmic Center Sun Yat‐Sen University Guangzhou China; ^5^ School of Optometry and Vision Sciences Cardiff University Cardiff UK

## INTRODUCTION

**Jeremy A. Guggenheim**

High‐prevalence diseases are generally attributed to a combination of genetic susceptibility and exposure to lifestyle‐related risk factors. In the case of myopia, at each end of this multifactorial aetiology spectrum are examples with a purely genetic or purely environmental origin. Pedigrees carrying rare, highly penetrant disease‐causing mutations can cause myopia irrespective of lifestyle risk factor exposure, while deprivation of form vision in early life leads to myopia across a wide range of genetic backgrounds. The focus of this Point‐Counterpoint article are children whose refractive error is not caused by these extremes: Is their myopia predominantly genetic or predominantly environmental?

This question really matters. Public health efforts need to address the root cause of myopia if they are to reverse the current epidemic. Should interventions be targeted at children—for example, in the form of drugs or optical devices personalised to the individual child? Or should interventions be targeted at the environment of all children—for example, by altering education systems or restricting the use of smartphones? Here, Ian Morgan and Virginie Verhoeven present the evidence that myopia is predominantly environmental or predominantly genetic.

## POINT

**Virginie J. M. Verhoeven**

Whether myopia is driven by genetic or environmental factors is a David versus Goliath discussion in many ways, with contrasting viewpoints competing to explain this complex condition. Here, I will outline the key evidence supporting the genetic basis of myopia, while disclosing my background in genetics, which may have shaped my perspective.

The myopia journey begins with genetics—we all carry a ‘backpack’ of genes inherited from our parents, determining our susceptibility to this condition. While environmental factors, such as near work or reduced outdoor activity, can shape the progression of myopia, they do so largely in the context of an existing genetic predisposition. Without the right genetic background, there is no foundation for myopia to develop.

First of all, it is the family tree that gives us the clearest glimpse into the likelihood of myopia development. Heritability is a statistical measure that estimates the proportion of variation in a trait within a population that can be attributed to genetic differences, rather than environmental factors. Heritability estimates for myopia and refractive error are striking, ranging from 60% to 90%, indicating that genetic factors account for the majority of variation, with only 10% to 20% attributable to environmental influences.^1^, ^2^ Family studies provide robust evidence. It is well‐established that if one or both parents are myopic, then the likelihood of the child developing myopia increases significantly.^3^, ^4^, ^5^, ^6^ Twin studies provide compelling additional evidence: Identical twins, who share 100% of their genes, show significantly higher concordance rates for myopia compared with fraternal twins, who share only 50% of their genes.^7^, ^8^, ^9^ As twins inherently grow up in similar environments, these findings suggest that genetic factors outweigh shared environmental influences.

We distinguish between common myopia, which results from a combination of genetic and environmental factors, and Mendelian (or syndromic) myopia, which is unequivocally driven by a single genetic mutation. In cases of Mendelian myopia, such as inherited retinal dystrophies or connective tissue disorders, environmental factors play little to no role.^10^, ^11^ These are by no means rare exceptions or black swans; recent research indicates that Mendelian forms of myopia occur far more frequently than previously thought, underscoring their significant contribution to the overall burden of myopia.^10^, ^11^ But also for common myopia, genetic predisposition remains a key determinant, with environmental factors modulating the expression of this genetic risk. Genome‐wide association studies (GWAS) in population‐based cohorts have demonstrated that everyone carries a certain level of genetic susceptibility to myopia, quantified by polygenic risk scores.^12^ These scores represent the accumulation of risk or protective factors across multiple genes, each contributing a small amount to the overall likelihood of developing myopia. For most individuals, environmental factors may influence the likelihood of developing myopia, but the underlying susceptibility or protection is fundamentally rooted in their genes. For instance, children with high polygenic risk scores are significantly more likely to develop myopia, even in environments with reduced near‐work activity or increased outdoor time.^13^ Conversely, Prof Morgan and I, despite being highly educated and engaging in significant near work throughout our careers, have not developed myopia. Based on environmental arguments, one might expect otherwise, but our genetic makeup has likely protected us. This underscores the critical role of genetics in determining susceptibility to myopia.

The myopia epidemic observed in East Asia provides a case study. Although the prevalence of myopia is rapidly increasing in Asian countries, there remains a small proportion of individuals who do not develop myopia.^14^ It is argued that the rapid rise in prevalence cannot be attributed to genetic factors, as gene pools remain stable over short periods.^15^ While this argument is valid, the increase in prevalence might as well reflect an amplification of genetic risk in response to changing environmental pressures, rather than the dismissal of genetic contributions.

The so‐called ‘missing heritability’ problem refers to the gap between heritability estimates from twin studies and the proportion explained by identified genetic variants. This gap is not unique to myopia but is a common challenge in complex traits.^16^ It reflects limitations in current genetic methodologies, such as the inability to capture rare variants, gene–gene and gene–environment interactions and epigenetic modifications. Advances in whole‐genome sequencing are expected to address these gaps, uncovering additional genetic contributors to myopia.

This brings me to the future and how to manage myopia. Genetics hold the key to effective therapies. It can and should inform personalised approaches to its prevention and treatment. Medical professionals need to consider family history more rigorously and offer genetic testing if considered appropriate, particularly in cases of high refractive error, a positive family history, poor response to therapy, or the presence of additional ocular or systemic features^10^, ^11^. Naturally, this applies only when genetic testing is available and accessible. Otherwise, simply asking parents if they have myopia or quickly drawing a family tree could serve as a valuable first step in predicting and managing myopia in their children. Although I believe it is incredibly important, for many reasons, I doubt that future generations will spend more time outdoors. With the intensifying heat in Asia and the growing addiction to screens and social media platforms, this seems increasingly unrealistic. However, by identifying children at high genetic risk, we can take early measures, such as increased outdoor time or limiting screen time, as well as myopia control, to slow the progression.^10^

To conclude, while environmental factors play a role in the progression of myopia, the evidence overwhelmingly supports genetics as the primary driver. The rising prevalence of myopia reflects an interaction between genetic predisposition and environmental triggers, not a dismissal of genetic contributions. As our understanding of myopia's genetic basis continues to grow, it offers a roadmap for more effective prevention and treatment strategies. It is the environment that shapes our world, but it is genetics that truly shape our vision.

## COUNTERPOINT

**Ian G. Morgan**

This debate is about the causes of variation in refractive status between individuals in a given population, at a given time. At one extreme, the hypothesis is that all variations are written into the genome. At the other extreme, the hypothesis is that all variation is driven by environmental exposures. My task is to put the case for the environmental hypothesis.

It is important to be clear about what the debate is. Some argue that because all living organisms are made up of cells that contain and are controlled by genes, living in an environment that can influence their operation, the question of genes or environment is absurd because life obviously depends on both. This argument is irrelevant because this debate is not about what is required for life. It is about the causes of variation.

It has a specific context—the epidemic of myopia that has emerged over the past 60–70 years in most parts of East Asia and Singapore in Southeast Asia.^15^, ^17^ In this region, defining the causes of variation is a very practical question. Over the past 60–70 years, the prevalence of myopia has risen from around 20%–30% in young adults to around 80%, and in parallel, 10%–30% of these cohorts have become highly myopic, putting them at high risk of subsequent uncorrectable visual impairment and blindness.^18^ The rise in the prevalence of high myopia has been disproportionately high relative to the rise in the prevalence of myopia,^19^, ^20^ so prevention and control have become a priority. Identifying the causes is important because if myopia in most people is written into the genome, it is likely to be hard to prevent, although the interventions that have been developed to control myopia progression may also prove to be useful in prevention.^21^, ^22^ In contrast, if myopia is due to causal environmental exposures, we can attempt to modify or remove those exposures without more invasive interventions.

Over the past 20–30 years, there has been a major change in our understanding of the aetiology of myopia. Only 20–30 years ago, it was widely believed that myopia in humans was strongly genetically determined and that environmental exposures had, at most, a very limited role to play or even none at all.^23^ As Sorsby put it, ‘there is cumulative, direct and incontrovertible evidence that myopia is genetically determined’. This hypothesis co‐existed with a large body of experimental evidence that visual experiences could significantly affect refractive development in animals,^24^, ^25^ but logically this did not require abandoning the dominant hypothesis about human myopia—it meant only that environmental effects were possible. But this evidence took on greater significance once the genetic hypothesis was invalidated.

The genetic hypothesis is completely inconsistent with the rapid emergence of an epidemic of myopia.^26^, ^27^ Gene pools simply do not change that fast, so the causal factors are almost certainly environmental. With environmental factors, change can be very rapid, particularly when change is induced by human social activity, such as the rapid development of mass education systems.

While the rapid emergence of an epidemic in itself rendered the genetic hypothesis unviable, it is also important to understand how the evidence that led to the dominance of the genetic hypothesis is not as conclusive as once thought. The most influential evidence in favour of genetic determination came from the high heritability of myopia in several twin studies.^7^, ^8^, ^9^ The logic is very simple and apparently powerful—monozygotic (MZ) twins are almost identical genetically, while dizygotic (DZ) twins share, on average, half their genes. So, if MZ twins are more concordant than DZ twins in a trait, ideally twice as concordant, this would suggest a genetic contribution to the trait. Myopia seemed to be a textbook example, with a high MZ concordance of 70%–90% and roughly twice the DZ concordance of 30%–45%.

However, this logic depends totally on the equal environment assumption; that members of MZ twin pairs are not more similar in their exposure to trait‐relevant factors than members of DZ twin pairs.^28^ If environmental factors contribute significantly to the difference in MZ and DZ concordance, the logic falls over. Consequently, a high heritability can sometimes point to a high genetic contribution, or at the limit, it can mean absolutely nothing. Moreover, in relation to myopia, it is known that MZ twins are more concordant in educational outcomes than DZ twins,^29^ which violates the common environment assumption for what is now known to be a major causal environmental influence on myopia.^30^, ^31^, ^32^ All this means that a high twin heritability does not prove or even strongly imply a genetic contribution.

Ultimately, validation of the heritability estimates can only come from identifying the associated molecular variation in the germline that explains the high heritability. In fact, extensive molecular genetic analysis has failed to find anything like sufficient associated molecular variation, with current estimates accounting for, at best, a heritability of around 20%,^1^, ^12^ with a theoretical upper limit of around 35%.^33^ This mismatch is in fact very common with complex human traits—so much so that the general term ‘missing heritability’ has been coined.^16^ A few molecular geneticists continue to argue that improved techniques and larger sample sizes will find the missing heritability, but, in the case of myopia, there is now such extensive documentation of causal environmental exposures for myopia that this hope seems doomed to be disappointed.

Two other arguments have been used to support significant genetic contributions. It has been argued that the fact that children with myopic parents are more likely to be myopic^34^ means that myopia is genetic, although this parental effect could, at least in principle, be attributed to myopic parents creating myopiagenic environments. Recent evidence suggests that the parental myopia effect involves both genetic and environmental factors.^35^ It is worth noting that most of the children who have become myopic during the development of the myopia epidemic have not had myopic parents. Arguments based on genetic predisposition therefore fail. If the parents had the predisposition, why did they not become myopic? But if the parents did not have the predisposition to pass on to their children, why did the children become myopic? It must be environmental factors that are making the difference.

The second argument has been that racial/ethnic differences in prevalence suggest that there is a genetic basis for the differences. This is wrong in principle since the genetic differences between humans are small, and little of this is associated with racial or ethnic differences.^36^ In contrast, environmental differences can be very marked, particularly differences in the social environment, such as differences in the level of development of education systems. In addition to these theoretical arguments, modern molecular genetic analysis has in practice failed to find significant differences in the single‐nucleotide polymorphisms (SNPs) associated with myopia between ethnic groups.^1^, ^12^ Thus, the three main pillars of the belief that myopia is largely genetic do not stand up to rigorous scrutiny.

In contrast to the failure of genetic explanations, there is a massive body of observational epidemiological evidence for the role of specific environmental factors. Two key environmental exposures have been identified.^37^ One is modern mass schooling. It was only after the end of the Second World War that education systems began to be introduced systematically in less developed parts of the world, and this is still a work in progress. The rapid development of mass highly competitive education systems in the Asian Tigers (Singapore, Hong Kong, Taiwan and South Korea) and the later exceptionally rapid development of similar education systems after the end of the Cultural Revolution in China have closely paralleled the development of the epidemics of myopia.^17^ Specific features of educational exposure in East Asia and Singapore that have led to the emergence of an epidemic of myopia, such as intensive education from an early age, heavy homework loads and high participation in additional tutorial classes out of school hours, have been identified.^38^

The impact of education also explains one of the most common observations in myopia research—that children become more myopic as they pass through higher levels of schooling. This is such a common observation—that it is often assumed that myopic shifts in refraction occur naturally as children get older. However, close examination of the epidemiological evidence does not support this assumption. In general, children who do not go to school rarely become myopic; children who receive little education develop little myopia, which associates duration of educational exposure with the development of myopia; and there is a link between years of education and the prevalence of myopia. This evidence has been reviewed in detail elsewhere.^17^

Recent evidence from Chinese studies also strongly links educational exposure with myopia.^31^, ^39^, ^40^, ^41^, ^42^, ^43^ The strict enrolment rules that apply in China mean that within one school grade, there are children differing by up to 12 months in age. The oldest children in one grade may be only a few days younger than the youngest in a higher grade, but they are very different in refractive status. Similarly, the younger children in one grade may be only a few days older than those in the next lower grade, but they are very different in refractive status. In general, a child's refraction correlates with the number of years that they have been at school, rather than with their age, and within a grade, on average, there is little difference between the youngest and the oldest children. These additional results suggest that most, if not all, of the myopic shift in refraction that occurs with age in children who attend school is driven by grade‐specific exposures, rather than by age itself.

Another general finding in the epidemiology of myopia is that it affects both boys and girls, with girls generally more myopic than boys. However, in Orthodox and Ultra‐Orthodox Jewish families in Israel, the prevalence of myopia in boys is as high as it is in both sexes in East Asia, but very much lower in their sisters.^44^, ^45^ This unexpected observation can be readily explained by the hypothesis that educational exposures play a major role in the development of myopia because boys receive an intensive education from an early age, whereas the education of girls in these religious families is much less intense. A genetic explanation cannot be excluded, given that rare sex‐linked forms of myopia are known,^46^ but the educational hypothesis provides a simple explanation that is consistent with all of the other data. It is interesting to note that a detailed examination of data from The Netherlands suggests that males once tended to be more myopic than girls, but in recent years, girls have become more myopic than boys,^47^ arguably because social restrictions on the education of girls have markedly decreased over the past 50 years. The ability of the hypothesis that education causes myopia to explain these diverse observations strengthens its credibility.

More direct evidence for causality generally requires randomisation to exposures, but most of the evidence in favour of causal impacts of education discussed above comes from observational studies, where randomisation does not occur. In relation to myopia and education, randomisation to different amounts of education would be clearly unethical, making most observations dependent on natural experiments. However, the causal nature of the associations has been assessed in a few cases. Using Mendelian randomisation,^31^ it has been shown that having SNPs associated with more years of schooling leads to more myopia, whereas having genes associated with myopia does not lead to more years of schooling. This establishes a causal link between more years of education to more myopia. Causality has also been assessed in relation to years of education and to annual educational exposures using regression discontinuity analysis, with positive results.^30^, ^32^, ^39^, ^40^, ^41^, ^42^

Thus, the conclusion that myopia is driven by environmental exposures that involve education is solidly based on a comprehensive pattern of associations between myopia and a range of aspects of education such as high levels of school attendance for up to 12 years and engagement in near‐work activities. Where more direct tests of causality have been possible, they have supported the existence of causal links from education to myopia.

The second key environmental exposure is the amount of time that children spend outdoors.^4^, ^48^, ^49^ In this case, intervention trials have been ethically approved, and a protective causal link between more time outdoors and less myopia has been demonstrated in intervention trials.^50^, ^51^, ^52^ Furthermore, myopia prevention based on increased time outdoors is being successfully implemented in practice.^53^, ^54^, ^55^

Having a plausible mechanism that could underpin a causal link provides strong support for a causal hypothesis.^56^ In relation to education and myopia, a clear mechanism has not been established, despite decades of speculation and research, although the demands of near work for reading and writing are commonly invoked. But how performing near work would lead to myopia is not clear. In the case of time outdoors, the hypothesis that the protective effect of time outdoors is due to the higher light intensities outdoors during daylight hours,^48^, ^57^ which result in more release of retinal dopamine and more inhibition of axial elongation, has received strong support from animal experimentation.^58^, ^59^ A recent hypothesis has suggested that the higher spatial frequency components of outdoor scenes that are markedly reduced indoors may also play a role,^60^ and preliminary reports suggest that a successful intervention trial has been performed (Weizhong Lan, personal communication).

In summary, genetic change cannot explain the rapid increase in prevalence and severity of myopia that has been seen in East Asia and Singapore. While evidence on changes in the prevalence of myopia in the rest of the world is much more limited, there also appears to have been an increase relative to a time when very few people received any formal education, and this, too, is unlikely to be explained by genetic change. Genetic differences do not explain the localisation of the epidemic of myopia, but they may contribute to the parental myopia effect. In contrast, the alternative environmental hypothesis, and specifically, the link of intensive education from an early age and limited time outdoors to the development of myopia seems to be consistent with the available data, explaining many detailed features of the evidence. Therefore, these environmental hypotheses currently provide the best framework for explaining the development of the predominant form of myopia in the world today, namely, axial myopia that develops in association with schooling, and this should remain the case unless future research comes up with solid contradictory evidence.

## SUMMARY

**Jeremy A. Guggenheim**

### Points of agreement

- Intensive education, insufficient time outdoors and, potentially, high levels of electronic device‐based near viewing play an important role in myopia development.
- Molecular genetic studies have identified rare genetic variants that cause high myopia in a deterministic manner, as well as hundreds of common genetic variants that have more subtle, non‐deterministic effects on the risk of myopia.
- Polygenic risk scores can explain only 20% of the variation in refractive error in the population, which falls far short of the 70%–90% heritability estimated from twin studies (this deficit is termed the ‘missing heritability’).

### Issues to be resolved

- Is the 70%–90% heritability observed in twin studies evidence of a strong genetic predisposition or an anomaly caused by a higher concordance of environmental risk factor exposure in monozygotic twin pairs compared to dizygotic twin pairs?
- Will larger genome‐wide association studies (GWAS), combined with rare variant analysis, gene‐gene interaction and gene‐environment interaction studies, explain the missing heritability of refractive error or have current polygenic risk scores already reached their limit?
- Are geographic differences in myopia prevalence mostly driven by lifestyle differences between countries or by genetic differences between populations?
- Is the increased risk attributed to parental myopia mostly due to genetics or to parents creating a myopiagenic environment for their children?
- How can conflicting findings be reconciled, such as within‐family differences in myopia risk associated with regular versus Orthodox Jewish schooling, or the absence of myopia in a proportion of children growing up in urban East and Southeast Asia despite a highly myopiagenic environment?

## FUNDING INFORMATION

This work was funded by UK Research and Innovation (UKRI) under the UK Government's Horizon Europe funding guarantee (grant number EP/Y032292/1). Funded by the European Union (Project 101119501—MyoTreat—HORIZON‐MSCA‐2022‐DN‐01). Views and opinions expressed are those of the author only and do not necessarily reflect those of the European Union or UKRI. Neither the European Union nor the granting authority can be held responsible for them.

## CONFLICT OF INTEREST STATEMENT

No financial conflicts of interest to declare.

## Data Availability

No data were generated as part of this study.
